# Correlation between Maximum Clot Firmness in FIBTEM and Fibrinogen Level in Critical Trauma Patients

**DOI:** 10.1155/2019/2756461

**Published:** 2019-11-22

**Authors:** Supphamongkhon Khunakanan, Osaree Akaraborworn, Burapat Sangthong, Komet Thongkhao

**Affiliations:** ^1^Department of Surgery, Faculty of Medicine, Prince of Songkla University, Hat Yai, Songkhla 90110, Thailand; ^2^Division of Trauma and Critical Care, Department of Surgery, Faculty of Medicine, Prince of Songkla University, Hat Yai, Songkhla 90110, Thailand

## Abstract

**Background:**

Both fibrinogen level and rotational thromboelastometry (ROTEM®) are accurate tests to demonstrate a bleeding tendency. FIBTEM® is one type of ROTEM test to determine the function of fibrinogen. An advantage of FIBTEM is helping physicians make proper decisions for blood component transfusions. However, the correlation between fibrinogen level and FIBTEM is still unclear.

**Objective:**

The aim of this study was to demonstrate a correlation between maximum clot firmness (MCF) in FIBTEM and fibrinogen level in critical trauma patients.

**Methods:**

Data were retrospectively collected from 87 patients who visited the emergency department between May 2017 and January 2019 in Songklanagarind Hospital. Blood specimens were sent for both ROTEM evaluation and fibrinogen level. The data were analysed with STATA program version 12.1.

**Results:**

Eighty-seven patients were enrolled in the study over the 21-month period. The patients consisted of 73 males (83.9%) with a median age of 40 years. Seventy-three patients (83.9%) were still alive. The following equation from FIBTEM MCF was used to predict fibrinogen level: fibrinogen level = 138 + (15.2 × FIBTEM MCF) (Lin's concordance correlation coefficient of 0.52, *P* < 0.001). The results showed a good correlation of FIBTEM MCF to predict patients with hypofibrinogenemia (area under ROC curve = 0.81). Patients with normal fibrinogen levels received significantly fewer units of all types of blood components.

**Conclusion:**

FIBTEM MCF had poor prediction of fibrinogen level; however, it can help to identify patients who have hypofibrinogenemia.

## 1. Introduction

Traumatic injuries were the 2nd leading cause of global death [1]. The mortality rate related to uncontrolled bleeding was about 40%. Trauma-induced coagulopathy is another factor of severe haemorrhage and uncontrolled bleeding. Trauma-induced coagulopathy was explained by four major triggers: haemodilution, hypothermia, acidemia, and hypoperfusion [2]. Early detection of trauma-induced coagulopathy can prevent the incidence of severe haemorrhage. In the past, a coagulogram test played a major role in detecting a bleeding tendency. However, this test had many limitations that included accuracy and wasted time.

Therefore, viscoelasticity testing was developed to resolve the limitations. Determination of viscoelasticity involves an investigation of the coagulation process that checks the clot from initiation to stabilization. Nowadays, there were two basic methods to determine viscoelasticity: TEG® (thromboelastography) and ROTEM® (rotational thromboelastometry). Fibrinogen is the substrate of fibrin, which is the beginning point of coagulation. It forms fibrin by coagulation factors and aggregates the platelets to strengthen the firmness of a blood clot. Recent evidence indicated that when the fibrinogen level was <2 g/L, there was a high risk of severe bleeding in postpartum haemorrhage patients [3]. The traditional method to measure fibrinogen level was the Clauss method which has a long turnaround time. ROTEM® is one type of viscoelasticity test. These technologies provided a visual assessment of clot formation and subsequent lysis under low shear conditions. FIBTEM is a kind of ROTEM which specifically studies the fibrinogen function by using cytochalasin D reagent to inhibit the platelet function. So, the clot formation was only contributed by the fibrinogen. Although fibrinogen level and FIBTEM were both markers of fibrinogen, their correlation was still unclear. A previous study was attempted to predict fibrinogen level from the FIBTEM maximum clot firmness (MCF) in trauma patients in a Western population, but it showed a poor correlation between FIBTEM MCF and fibrinogen level [4]. However, there is no study about this correlation created in Southeast Asian population especially in Songklanagarind Hospital.

Therefore, the aim of this study was to demonstrate the correlation between the MCF in FIBTEM and fibrinogen level in critical trauma patients in Songklanagarind Hospital.

## 2. Materials and Methods

This was a retrospective cohort study conducted in Songklanagarind Hospital which is a trauma centre of Southern Thailand. All adult patients who were identified as age >15 years and met the trauma team activation criteria were included in the study. Patients whose fibrinogen level or ROTEM was not obtained were excluded.

### 2.1. Fibrinogen Level

Blood specimens of at least 2.7 mL were obtained from the patients right after the patients arrive at the emergency department and were collected in 3.2% sodium citrate tubes. The process of measurement started when the blood specimen reached the hematology laboratory. The specimens were centrifuged for separation of the plasma and red blood cells (Sorvall ST 100 16R). After plasma was obtained by the centrifuge process, the fibrinogen level was measured. Measurement of fibrinogen level in Songklanagarind Hospital was performed using Thromborel® S Reagent and the appropriate assay on a SYSMEX 1500® or SYSMEX 2500® coagulation analyser. The fibrinogen concentration may be derived by analysing the change in optical signal during the prothrombin time determination using a derived fibrinogen calibration curve. The total process time was not more than 1 h from the time the blood reached the laboratory. The study classified patients into two groups according to the fibrinogen level: the hypofibrinogenemia group was defined as the fibrinogen level <200 mg/dL and the other group was the normal fibrinogen level group.

### 2.2. FIBTEM MCF

The blood specimens sent for FIBTEM also required at least 2.7 mL and were collected in 3.2% sodium citrate tubes. After reaching the laboratory, the specimens were incubated at 37°C for 5 min. The specimens were then mixed with the specific reagents, and the process began. The process took at least 30 min to analyse the data from the ROTEM. The total process time was about 1 h. In the FIBTEM test, the fib-tem® solution neutralizes the thrombocytes *in vitro* with cytochalasin D and contains a recalcification agent. In the thromboelastometric measurement, the clotting is continuously monitored by the ROTEM® analyser. There is an automatic calculation for the clotting time (CT), clot formation time, alpha angle, and clot amplitude after 10 minutes and MCF. These parameters describe haemostasis from clot activation, clot formation, clot polymerization, clot stability, and clot lysis.

### 2.3. Statistics

From a review of the literature, the correlation coefficient between fibrinogen level and the FIBTEM MCF was obtained from a study by Meyer et al. [5]. The correlation coefficient was 0.64, and after calculation with a sample size formula, the final sample size was 40 patients, but we included a total of 87 patients to improve the power of the study. The data record tables were fulfilled by reviewing the inpatient department medical record computer program. The statistical analysis was performed with the STATA program (version 12.1). Nonparametric data are reported as median and interquartile ranges (IQR). Linear correlation was analysed using the Pearson method. A receiver operating characteristic curve analysis was performed to evaluate the performance of the ROTEM instrument with respect to discriminating patients with admission fibrinogen levels. Linear regression was used to assess the correlation between ROTEM instrument factors and fibrinogen level. A *P* value less than 0.05 was chosen to represent statistical significance throughout. The study was approved by the Prince of Songkla University, Human Research Ethics Committee, Faculty of Medicine.

## 3. Results

Eighty-seven patients were enrolled into the study over the 21-month period. The median (IQR) age of the patients was 40 (17–65) years. Most patients were males. The demographics and Lin's concordance correlation coefficient was used to demonstrate the correlation of predicted fibrinogen level from FIBTEM MCF and fibrinogen level. Characteristics of all patients are shown in Table 1.

### 3.1. ROTEM Characteristics between Normal Fibrinogen Level Group and Hypofibrinogenemia Group

**Figure 1:**
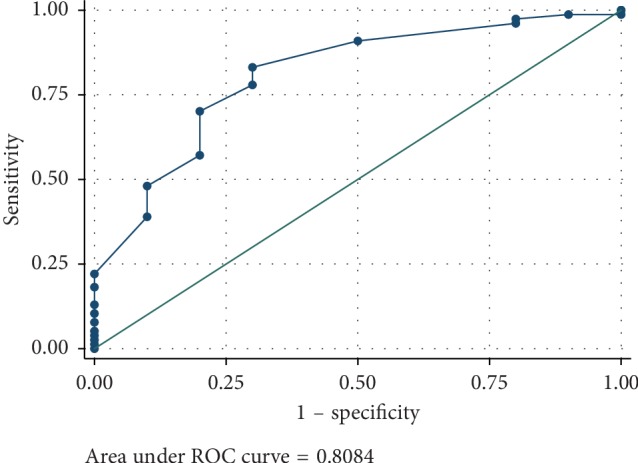
Area under ROC curve of FIBTEM MCF to predict the hypofibrinogenemia state.

The data analysis showed significant differences in the EXTEM and FIBTEM parameters between the normal fibrinogen level group and hypofibrinogenemia patient groups. The patients with higher fibrinogen level had shorter clotting time initiation in EXTEM (61 vs 109 sec; *P* < 0.01) and had higher amplitude of clot firmness in EXTEM MCF (60 vs 50 mm; *P* < 0.01). Likewise, the patients with higher fibrinogen level had higher amplitude of clot firmness in FIBTEM MCF (12 vs 7.5 mm; *P* < 0.01) and higher amplitude in 10 min of FIBTEM (11 vs 6 mm; *P* < 0.01) (Table 2). Moreover, the good diagnostic performance of FIBTEM MCF in predicting hypofibrinogenemia is summarized in Figure 1 using the area under ROC curve (AUC = 0.81).

### 3.2. Correlation between Fibrinogen Level and FIBTEM MCF

**Figure 2:**
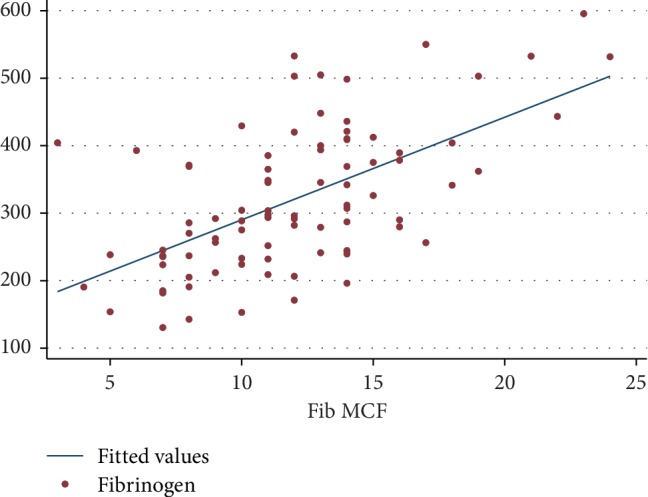
Linear regression between fibrinogen level and FIBTEM MCF.

The correlation between fibrinogen level and FIBTEM MCF was the primary objective of this study, and linear regression analysis was performed. It was determined that the correlation of both parameters can be calculated from an equation: fibrinogen level = 138 + (15.2 × FIBTEM MCF). The correlation of the predicted value of fibrinogen level and the actual value was demonstrated by Lin's concordance correlation coefficient of 0.52 (*P* < 0.001). The linear regression plot is shown in Figure 2.

**Figure 3:**
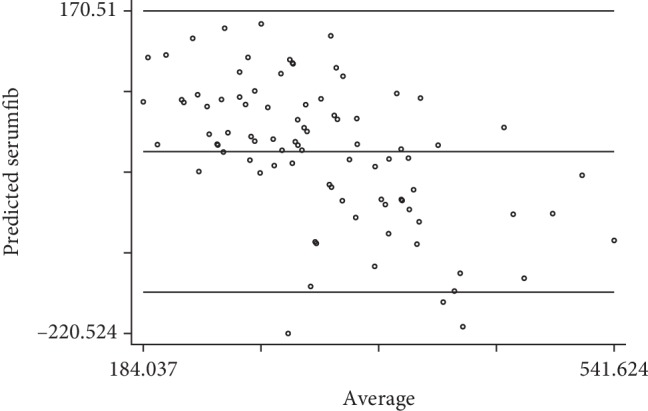
The Bland–Altman plot between predicted values and actual values of fibrinogen level.

The possible error of this equation was further demonstrated by the Bland–Altman plot between the predicted value of serum fibrinogen and the actual serum fibrinogen level.  Figure 3 shows that if the fibrinogen level is quite high, the predicted value of fibrinogen level would be underestimated (error was minus). On the other hand, if the fibrinogen level was quite low, the predicted value of fibrinogen level would be overestimated.

### 3.3. Correlation between Volume of Transfused Blood Component and Level of Fibrinogen Level

The relationship between the normal and the hypofibrinogenemia patients group was analysed and was found that the normal patient group had significantly fewer units of transfused packed red cells (PRC) (1 unit vs 5 units; *P* < 0.01), fresh frozen plasma (FFP) (2 units vs 5 units; *P* < 0.02), and platelet concentrate (PC) (3 units vs 9.7 units; *P* < 0.02). The data are shown in Table 3.

## 4. Discussion

Our study found that FIBTEM MCF was able to predict hypofibrinogenemia state (area under ROC curve = 0.81), but it was not able to predict the actual level of fibrinogen level (Lin's concordance correlation coefficient of 0.52). This finding is consistent with the previous study by Rourke et al. [4]. Their study evaluated the ability of ROTEM analysis to assess the fibrinogen levels in acute traumatic coagulopathy patients, and they found that the FIBTEM MCF measurements were significantly correlated with Clauss fibrinogen levels. However, the *R*^2^ from their study was 0.27 which was not a good correlation as well. From a literature review, only one study was interested in the correlation between FIBTEM MCF and fibrinogen level in trauma patients. The study by Meyer et al. [5] had similar criteria to those in our study for the enrolment of trauma patients but that study was conducted in London, UK. This is the major difference between their study and our Asian population. Meyer et al. analysed the linear regression between fibrinogen level g/dL and FIBTEM MCF (mm) measured on arrival in 182 trauma patients. They found that the following equation could be representative:

(1) $$\begin{array}{c} \text{FIBTEM MCF }\left(\text{mm}\right)=4.47\times \left(\text{fibrinogen level }\left[\text{g}/\text{L}\right]\right)+2.84. \end{array}$$

Their study as well as our study showed a poor correlation. (*R*^2^ = 0.41). The FIBTEM assay measures elasticity of the fibrin-based clot, which is dependent not only on fibrinogen but also on other proteins such as Factor XIII [6]. Colloid therapy (e.g., hydroxyethyl starch [HES]) may be another confounder because the presence of HES reduces FIBTEM values but increases Clauss fibrinogen concentration [7]. Fibrinogen is well known as the substrate of clot formation. It plays the major role in haemostasis pathway. Traditionally, fibrinogen was only supplemented when the levels fell below 100 mg/dL [8]. More recent evidence suggests that patients with fibrinogen levels <200 mg/dL have worse outcomes [3] which correlated with our study in which the group that had hypofibrinogenemia received more blood transfusions. Furthermore, other studies, including Meyer et al. [5], reported higher transfusion rates in hypofibrinogenemia patients. They indicated increased odds ratios for PRC, FFP, and PC transfusions at fibrinogen levels <2.5 g/L.

According to Miesbach et al. [9], plasma fibrinogen level from Clauss assay is better than prothrombin time-derived method in diagnosing or treating patients with low fibrinogen levels because the prothrombintime-derived method may potentially pose a greater risk to patients, as the plasma concentration may be erroneously reported as normal.

This study has some limitations. This study was retrospective in nature, and the number of patients enrolled in the study was quite low. However, based on the calculated sample size, the number of patients was probably sufficient to test the hypothesis. Moreover, sometimes there was difficulty obtaining blood samples because of severe traumatic injuries and rush situations followed by death. Therefore, many patients matched the trauma activation criteria but since their blood specimens were inadequate, they had to be excluded from the study.

## 5. Conclusions

FIBTEM MCF had poor prediction of accurate fibrinogen level. However, it can guide us to identify patients with hypofibrinogenemia.

## Figures and Tables

**Table 1:** Demographic data.

Age (years), median (IQR)	40 (17–65)

Gender	
Male	73 (83.9)
Female	14 (16.1)

BMI (kg/m^2^), mean (SD)	23 (4.2)

Mechanism of injury	
Motorcycle collision	61 (71.7)
Gunshot wound	2 (2.3)
Stabbed wound	9 (9.2)
Fall from height	5(5.7)
Car accident	6 (6.9)
Pedestrian collision	2 (2.3)
Others	2 (2.3)

ISS, median (IQR)	22 (14–33)

PS score, median (IQR)	0.94 (0.7–0.99)

Survived	
Yes	73 (83.9)
No	14 (16.1)

Fibrinogen level	
Level <200 mg/dL	10 (11.0)
Level >200 mg/dL	77 (89.0)

Data are reported as *n* (%) unless indicated otherwise. IQR, interquartile range; BMI, body mass index; SD, standard deviation; ISS, injury severity score; PS, physical status.

**Table 2:** FIBTEM characteristics between normal fibrinogen level and hypofibrinogenemia.

Variables	Median when fibrinogen level <200 mg/dL	Median when fibrinogen level >200 mg/dL	*P* value
FIBTEM			
MCF (mm)	7.5	12	<0.01
CT (sec)	45	44	0.05
A10 (mm)	6	11	<0.01

Data are presented as median. MCF: maximum clot firmness; CT: clotting time; A10: clot amplitude at 10 min.

**Table 3:** Correlation between volume of transfused blood component and fibrinogen level.

Blood components	Transfused units	*P*value
PRC		<0.01
Fibrinogen level <200 mg/dL	5 (1–10)	
Fibrinogen level >200 mg/dL	1 (0–5)	

FFP		<0.01
Fibrinogen level <200 mg/dL	8 (2–9)	
Fibrinogen level >200 mg/dL	2 (0–6)	

PC		0.02
Fibrinogen level <200 mg/dL	6 (0–10)	
Fibrinogen level >200 mg/dL	0 (0–6)	

Data are reported as median (IQR). PRC, packed red cells; FFP, fresh frozen plasma; PC, platelet concentrate.

## Data Availability

To access further available information of this study please contact: aosaree@gmail.com.
